# Supplementary material for: Hyperbolic photonic topological insulators
Source: Nat Commun. 2024 Feb 22;15:1647. doi: 10.1038/s41467-024-46035-y (PMC10884020; doi:10.1038/s41467-024-46035-y)
Supplement: Supplementary file 1 — Supplementary information [file 41467_2024_46035_MOESM1_ESM.pdf]

Supplementary Information : Hyperbolic photonic topological insulators

Lei Huang et al.

Supplementary Note 1. Numerical results on the spatial profile and robust one-way propagation of topological edge states in the face-centered hyperbolic lattice model. In this part, we present numerical results on the eigenvalue and robust edge propagation in the face-centered hyperbolic lattice model. Fig. S1(a) presents numerical results of the calculated eigenspectrum with φ=π. The color map represents the localization strength of eigenstates on lattice sites at the third layer (the same to Fig. 1(d) in the main text). The associated spatial distributions of eigenstates with different eigenenergies are plotted in Fig. S1(b). It is shown that the probability amplitudes are concentrated in the bulk region for two trivial bulk states with n=1 and n=121. And, two topological edge states with n=124 and n=147 exhibit the significant edge localizations.

In addition, it is widely known that one-way edge states, which are robust against defects, should exist in the energy region with nontrivial real-space Chern numbers. The coupled model equations of the model can be expressed as

$$i\frac{dc_m(t)}{dt} = \sum_n H_{mn}c_n(t) + \chi\psi_{\text{in}}(t) \quad , \tag{1}$$

where $c_m(t)$ is the probability amplitude at site m and H_{mn} represents the complex coupling between m-th and n-th sites. χ is the coupling rate of the input channel and $\psi_{in}(t)$ is the input signal. In the following, we always set $\chi = 1$. The input wave packet (at m=12) is given by $\psi_{in}(t) = exp(-(t-t_0)^2/100)\sin(\varepsilon_c t)$ with $\varepsilon_c = 0$ and $t_0 = 20$.

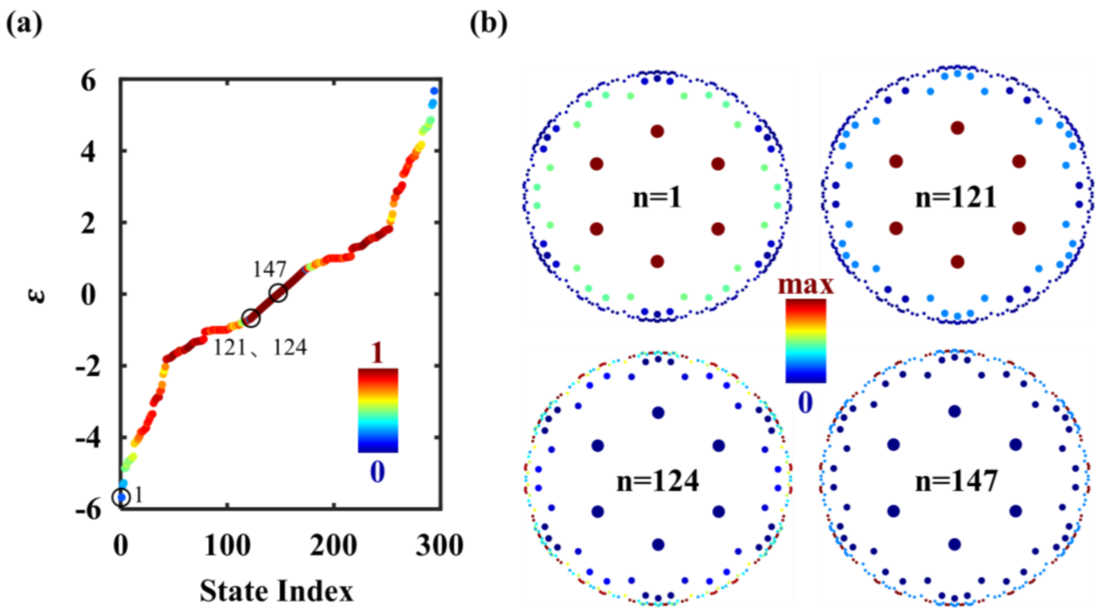

Fig. S1. (a) The eigenspectra of the three-layer face-centered hyperbolic lattice model with φ equaling to π. (b) Spatial distributions of hyperbolic eigenstates with n=1, 121, 124, 147. Top and bottom images correspond to bulk and edge states, respectively.

Fig. S2(a) shows the variation of the wave function in the time domain for the defect-free structure. The abscissa axis represents the label of lattice sites, and the ordinate axis represents the time. Fig. S2(b) shows the spatial distribution with t=20, 60 and 65. It can be seen that the input wave propagates unidirectionally along the boundary, manifesting the topological property of hyperbolic edge states. Next, we introduce the boundary defect by delating all NN, NNN, and NNNN couplings of a boundary site (m=64). Figs. S2(c)-2(d) display numerical results of the wave propagation in the defective hyperbolic lattice model. It can be observed that the input signal can bypass the defect without any backscattering, showing that our designed hyperbolic lattice model possesses topologically protected one-way edge states.

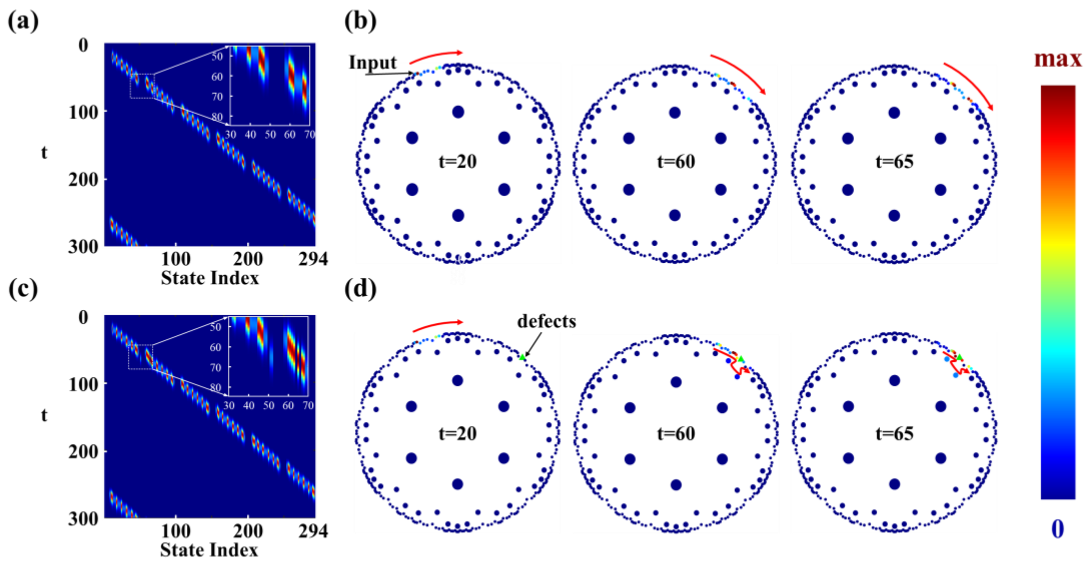

Fig. S2. (a) and (c). The variation of the wave function in the time domain with and without defect hyperbolic lattice model. (b) and (d). Spatial distributions at different times with and without defect hyperbolic lattice models.

Supplementary Note 2. The derivation of the effective tight-binding Hamiltonian of coupled ring resonators. It has been demonstrated that the momentum-space Hamiltonian of a lattice model with the translational symmetry can be mapped to the transfer matrix of coupled optical ring resonators. In this case, the real space Hamiltonian can be easily obtained by performing Fourier transform on the k-space Hamiltonian[1,2]. However, such a method is no longer hold in systems without Abelian translational symmetries. Therefore, it is necessary to provide a more general method to derive the effective tight-binding Hamiltonian of coupled ring resonators in hyperbolic lattices.

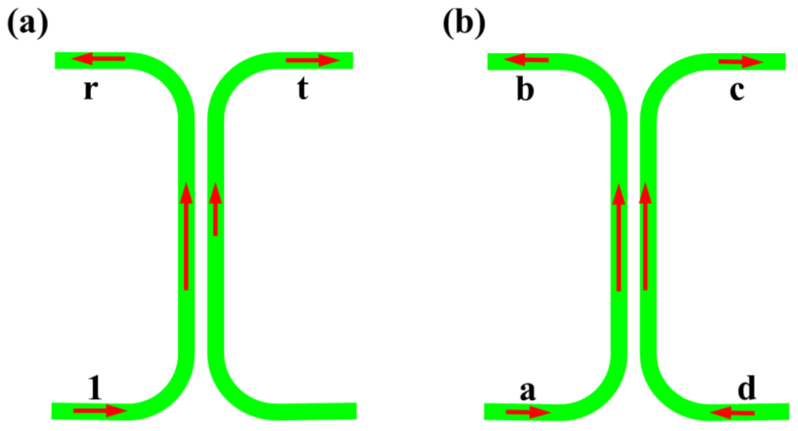

Fig. S3. The relationship between waveguide couplings. (a) Transmission situation when light is incident from the lower left corner. *r* is the reflection coefficient. *t* is the transmission coefficient. (b) Scattering matrix diagram when light passes through the waveguide coupler.

Firstly, we illustrate the coupling behavior between two nearby waveguides. When two waveguides are close to each other, as shown in Fig. S3(a), the electromagnetic wave can couple from one waveguide to the other. In this case, we assume that the amplitude of the injected wave is 1. After passing through the waveguide coupler, the wave amplitude in the injected waveguide is *r*, and the wave amplitude coupled to the other waveguide is *t*. Here, the backward scattering and losses in two waveguides are neglected, making the coupling between two spin modes (clockwise and counterclockwise eigenmodes of each site rings) become zero. Therefore, according to the law of energy conservation, we have $|r|^2 + |t|^2 = 1$. For simplicity, we represent *r* and *t* with a single parameter θ as $r = cos\,\theta$, $t = -i\,sin\,\theta$. Duo to the reciprocity, the light entering the waveguide coupler from the other direction has the same transmittance and reflectance. Therefore, the light passing through the coupler has the following relationship

$$\begin{bmatrix} b \\ c \end{bmatrix} = \begin{bmatrix} r & t \\ t & r \end{bmatrix} \begin{bmatrix} a \\ d \end{bmatrix} = \begin{bmatrix} cos\,\theta & -i\,sin\,\theta \\ -i\,sin\,\theta & cos\,\theta \end{bmatrix} \begin{bmatrix} a \\ d \end{bmatrix}, \tag{2}$$

where a, b, c and d correspond to wave amplitudes of four ports, as shown in Fig. S3(b). In this case, the scattering matrix of two coupled waveguides is represented by

$$\hat{S}(\theta) = \begin{bmatrix} cos\,\theta & -i\,sin\,\theta \\ -i\,sin\,\theta & cos\,\theta \end{bmatrix}. \tag{3}$$

Using the above proposed scattering matrix, the scattering equation of two coupled site rings through a single linking ring (as shown in Fig. S4) in the counterclockwise-spin subspace can be described by

$$\begin{bmatrix} a_1' \\ s_1' \end{bmatrix} = \hat{S}(\theta) \begin{bmatrix} a_1 \\ s_1 \end{bmatrix}$$
$$\begin{bmatrix} a_2' \\ s_2' \end{bmatrix} = \hat{S}(\theta) \begin{bmatrix} a_2 \\ s_2 \end{bmatrix} \tag{4}$$

where the amplitudes at different ports satisfy the relationships of $a_1' = a_1 e^{-i\xi}$, $s_1' = s_2 e^{-i(\Phi+\phi)}$,$a_2' = a_2 e^{-i\xi}$, and$s_2' = s_1 e^{-i(\Phi-\phi)}$ with ξ and $\Phi \pm \varphi$ being the propagation phases as marked in Fig. S4. It is noted that the propagation phases and the parameter θ satisfy the following relationships with $\xi = 2\pi\delta v/FSR_a$,$\Phi = \pi + 2\pi\delta v/FSR_s$, and $\theta = \sqrt{4\pi J/FSR_a}$, where $FSR_a \sim 1/L_a$ and FSR_s represents the free spectral range of the site ring and the linking ring. δv represents the difference between the operating frequency and the resonant frequency of the site ring. Combining the Eq. S3 and Eq. S4, we can get

$$\begin{cases} a_2 \sin\xi = a_1 e^{-i\phi}(\sin\Phi + \sin(\xi-\Phi)\cos\theta) \\ a_2 e^{i\phi}(-\sin\Phi\cos\theta + \sin(\xi+\Phi)) = a_1 \sin\xi\cos\theta \end{cases} \tag{5}$$

Here, we assume that the system stays in the weak coupling condition and possesses a small frequency shift, that is $4\pi J/FSR_a \sim 2\pi\delta v/FSR_a \sim \pi\delta v/FSR_s \sim \eta$ with η being a small parameter. Therefore, Eq. S5 can be simplified approximately to

$$\begin{cases} a_2\delta v = a_1 e^{-i\phi} J \\ a_2 e^{i\phi} J = a_1 \delta v \end{cases}. \tag{6}$$

Eq. S6 can be further written into a matrix form as

$$\begin{bmatrix} 0 & Je^{i\phi} \\ Je^{-i\phi} & 0 \end{bmatrix}\begin{bmatrix} a_1 \\ a_2 \end{bmatrix} = \delta v \begin{bmatrix} a_1 \\ a_2 \end{bmatrix}, \tag{7}$$

where the effective Hamiltonian is written as

$$\hat{H} = \begin{bmatrix} 0 & Je^{i\phi} \\ Je^{-i\phi} & 0 \end{bmatrix} \tag{8}$$

with δv being the effective eigenvalue. From above results, we can see that the coupling strength between two site rings is determined by the coupling angle of the scattering matrix. And, the coupling phase is determined by the propagation phase of ϕ. Thus, as illustrated in Figs. S4(c) and S4(d), we can tune the coupling strength by adjusting the separation distance between site rings and the link ring, while tuning of coupling phase by altering the vertical position of the link ring.

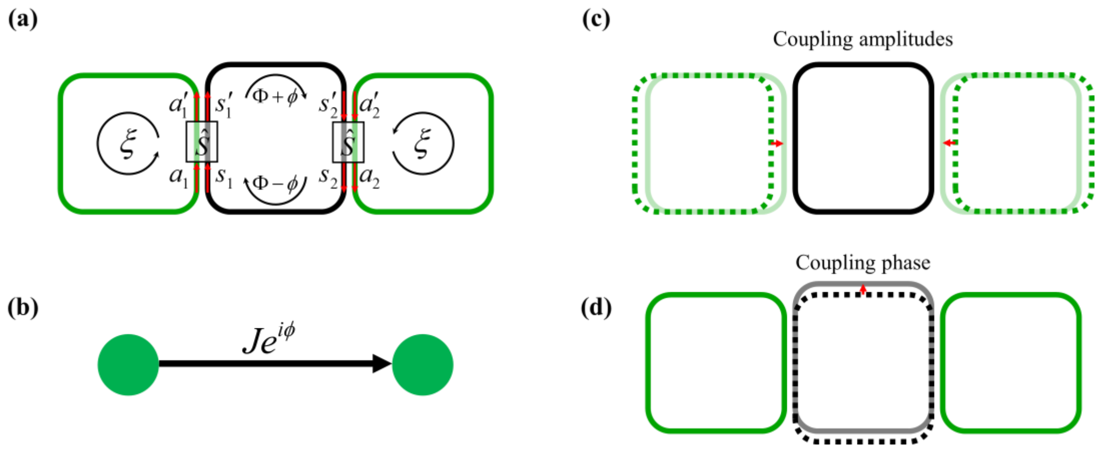

Fig. S4. (a)The illustration of two coupled site rings through a linking ring. a_1, a_2 are used to describe the intensity of light in the site ring, s_1, s_2 is used to describe the intensity of light in the link ring. ξ represents the phase of light after one loop in the site ring, Φ represents the phase of light after half a loop in the linking ring, and ϕ represents the phase difference caused by the coupling region being offset from its symmetric position. (b) Schematic diagram of lattice coupling. (c) Schematic diagram illustrating the tune of coupling amplitudes. (d) Schematic diagram illustrating the tune of coupling phase.

Next, we focus on the derivation of the effective Hamiltonian of six site resonators coupled by a single linking ring, as shown in Fig. S5. The scattering matrix of each site ring coupled to the linking ring is expressed as

$$\begin{bmatrix} a_i' \\ s_i' \end{bmatrix} = \begin{bmatrix} \cos\theta & -i\sin\theta \\ -i\sin\theta & \cos\theta \end{bmatrix} \begin{bmatrix} a_i \\ s_i \end{bmatrix} \text{ with } i = 1,2,3,\cdots,6 \tag{9}$$

with $a_i' = a_i e^{-i\xi}$,$s_i' = s_{i+1} e^{-i\Phi_i}$. We expand two scattering matrixes of the *i*-th and *i*+1-th rings as

$$\begin{cases} is_i \sin\theta = a_i(\cos\theta - e^{-i\xi}) \\ is_{i+1} e^{-i\Phi_i} \sin\theta = a_i \sin^2\theta + is_i \sin\theta\cos\theta \\ is_{i+1}\sin\theta = a_{i+1}(\cos\theta - e^{-i\xi}) \\ is_{i+2} e^{-i\Phi_{i+1}} \sin\theta = a_{i+1}\sin^2\theta + is_{i+1}\sin\theta\cos\theta \end{cases} . \tag{10}$$

It is clearly shown that, by eliminating the co-amplitudes of s_{i+1} and s_i, the amplitudes of a_i and a_i satisfy the following relationship of

$$a_{i+1}(\cos\theta - e^{-i\xi})e^{-i\Phi_i} = a_i(1 - \cos\theta\, e^{-i\xi}) \tag{11}$$

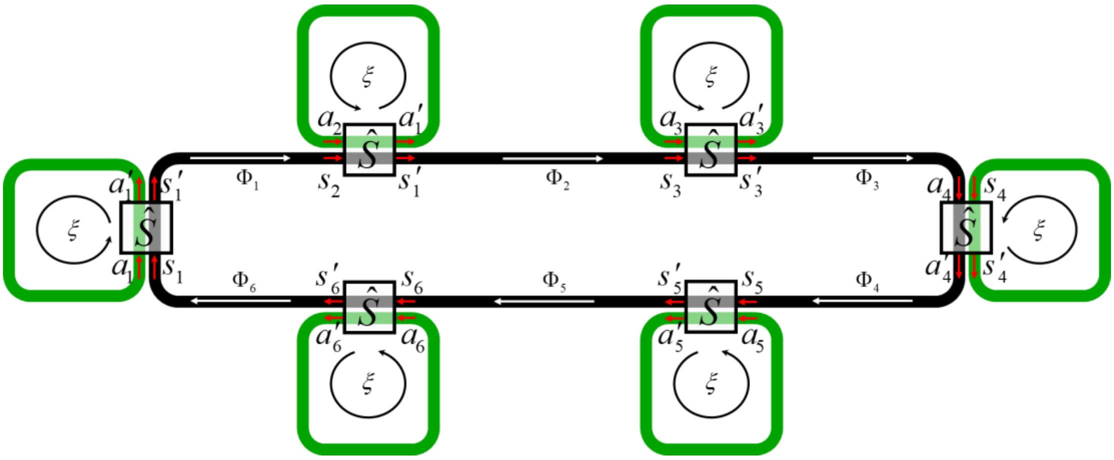

Fig. S5. The amplitude relationship of a link ring connected 6 site rings. a_i represents the amplitude of the wave from the i-th input point entering the coupling region, a_i' represents the amplitude of the wave from the *i*-th output point exiting the coupling region. ξ represents the phase accumulated when the light circulates around the *i*-th site rings. s_i represents the amplitude of the wave entering the coupling region from the linking ring to the *i*-th input point. s_i' represents the amplitude of the wave exiting the coupling region from the *i*-th output point to the linking ring. Φ_i represents the phase accumulated by the wave after leaving the coupling region of the *i*-th output point and before entering the coupling region of the (*i*+1)-th input point in the linking ring.

Eq. S11 can be re-expressed as

$$a_i\left(i\sin\frac{\xi}{2} + \sin^2\frac{\theta}{2}e^{-i\xi/2}\right) = a_{i+1}\left(i\sin\frac{\xi}{2} + \sin^2\frac{\theta}{2}e^{-i\xi/2}\right)e^{-i\Phi_i} - 2a_{i+1}\sin^2\frac{\theta}{2}\cos\frac{\xi}{2}e^{-i\Phi_i} \tag{12}$$

Using Eq. S12, we can obtain following six equations (with *i*=1, 2, 3, 4, 5, 6) as

$$\begin{cases} a_1\left(i\,sin\frac{\xi}{2}+sin^2\frac{\theta}{2}e^{-i\xi/2}\right)=a_2\left(i\,sin\frac{\xi}{2}+sin^2\frac{\theta}{2}e^{-i\xi/2}\right)e^{-i\Phi_1}-2a_2\,sin^2\frac{\theta}{2}cos\frac{\xi}{2}e^{-i\Phi_1} \\ a_2\left(i\,sin\frac{\xi}{2}+sin^2\frac{\theta}{2}e^{-i\xi/2}\right)=a_3\left(i\,sin\frac{\xi}{2}+sin^2\frac{\theta}{2}e^{-i\xi/2}\right)e^{-i\Phi_2}-2a_3\,sin^2\frac{\theta}{2}cos\frac{\xi}{2}e^{-i\Phi_2} \\ a_3\left(i\,sin\frac{\xi}{2}+sin^2\frac{\theta}{2}e^{-i\xi/2}\right)=a_4\left(i\,sin\frac{\xi}{2}+sin^2\frac{\theta}{2}e^{-i\xi/2}\right)e^{-i\Phi_3}-2a_4\,sin^2\frac{\theta}{2}cos\frac{\xi}{2}e^{-i\Phi_3} \\ a_4\left(i\,sin\frac{\xi}{2}+sin^2\frac{\theta}{2}e^{-i\xi/2}\right)=a_5\left(i\,sin\frac{\xi}{2}+sin^2\frac{\theta}{2}e^{-i\xi/2}\right)e^{-i\Phi_4}-2a_5\,sin^2\frac{\theta}{2}cos\frac{\xi}{2}e^{-i\Phi_4} \\ a_5\left(i\,sin\frac{\xi}{2}+sin^2\frac{\theta}{2}e^{-i\xi/2}\right)=a_6\left(i\,sin\frac{\xi}{2}+sin^2\frac{\theta}{2}e^{-i\xi/2}\right)e^{-i\Phi_6}-2a_5\,sin^2\frac{\theta}{2}cos\frac{\xi}{2}e^{-i\Phi_5} \\ a_6\left(i\,sin\frac{\xi}{2}+sin^2\frac{\theta}{2}e^{-i\xi/2}\right)=a_1\left(i\,sin\frac{\xi}{2}+sin^2\frac{\theta}{2}e^{-i\xi/2}\right)e^{-i\Phi_1}-2a_1\,sin^2\frac{\theta}{2}cos\frac{\xi}{2}e^{-i\Phi_6} \end{cases} \quad (13)$$

Combing these six equations, we have

$$a_1\left(i\,sin\frac{\xi}{2}+sin^2\frac{\theta}{2}e^{-i\xi/2}\right)=a_1\left(i\,sin\frac{\xi}{2}+sin^2\frac{\theta}{2}e^{-i\xi/2}\right)e^{-i\sum_{j=1}^{6}\Phi_j}-2a_1\,sin^2\frac{\theta}{2}cos\frac{\xi}{2}e^{-i\sum_{j=1}^{6}\Phi_j}$$

$$-2a_6\,sin^2\frac{\theta}{2}cos\frac{\xi}{2}e^{-i\sum_{j=1}^{5}\Phi_j}-\cdots-2a_3\,sin^2\frac{\theta}{2}cos\frac{\xi}{2}e^{-i(\Phi_1+\Phi_2)}-2a_2\,sin^2\frac{\theta}{2}cos\frac{\xi}{2}e^{-i\Phi_1} \quad (14)$$

For the sake of simplicity, we make the following substitutions with $\sum_{j=1}^{m-1}\Phi_j=\Phi/2-\varphi_{1m}$, $\sum_{j=m}^{N}\Phi_j=\Phi/2-\varphi_{m1}=\Phi/2+\varphi_{1m}$, where $\Phi=\sum_{j=1}^{N}\Phi_j$ is the phase for the light goes through the entire ring. Then, the above equation can be simplified as

$$a_1\left(i\,sin\frac{\xi}{2}+sin^2\frac{\theta}{2}e^{-i\xi/2}\right)=a_1\left(i\,sin\frac{\xi}{2}+sin^2\frac{\theta}{2}e^{-i\xi/2}\right)e^{-i\Phi}-2a_1\,sin^2\frac{\theta}{2}cos\frac{\xi}{2}e^{-i\Phi}$$

$$-2a_6\,sin^2\frac{\theta}{2}cos\frac{\xi}{2}e^{-i(\Phi/2-\varphi_{1,N})}-\cdots-2a_3\,sin^2\frac{\theta}{2}cos\frac{\xi}{2}e^{-i(\Phi/2-\varphi_{1,3})}-$$

$$2a_2\,sin^2\frac{\theta}{2}cos\frac{\xi}{2}e^{-i(\Phi/2-\varphi_{1,2})} \quad (15)$$

Similar to the above case with two coupled site rings, Eq. S15 can be expressed as

$$\delta v a_1=Ja_6e^{i\varphi_{1,6}}+\cdots+Ja_3e^{i\varphi_{1,3}}+Ja_2e^{i\varphi_{1,2}}, \quad (16)$$

when the system is in the weak coupling condition and possesses a small frequency shift.

Similar to the case of a_1, the amplitudes at other onsite rings can also be expressed as

$$\delta v a_i=Ja_6e^{i\varphi_{i,6}}+\cdots+Ja_{i+1}e^{i\varphi_{i,i+1}}+Ja_{i-1}e^{i\varphi_{i,i-1}}+\cdots+Ja_2e^{i\varphi_{i,2}}+Ja_1e^{i\varphi_{i,1}} \quad (17)$$

Writing Eq. S17 into a matrix form, we have

$$\begin{bmatrix} 0 & Je^{i\varphi_{1,2}} & Je^{i\varphi_{1,3}} & Je^{i\varphi_{1,4}} & Je^{i\varphi_{1,5}} & Je^{i\varphi_{1,6}} \\ Je^{i\varphi_{2,1}} & 0 & Je^{i\varphi_{2,3}} & Je^{i\varphi_{2,4}} & Je^{i\varphi_{2,5}} & Je^{i\varphi_{2,6}} \\ Je^{i\varphi_{3,1}} & Je^{i\varphi_{3,2}} & 0 & Je^{i\varphi_{3,4}} & Je^{i\varphi_{3,5}} & Je^{i\varphi_{3,6}} \\ Je^{i\varphi_{4,1}} & Je^{i\varphi_{4,2}} & Je^{i\varphi_{4,3}} & 0 & Je^{i\varphi_{4,5}} & Je^{i\varphi_{4,6}} \\ Je^{i\varphi_{5,1}} & Je^{i\varphi_{5,2}} & Je^{i\varphi_{5,3}} & Je^{i\varphi_{5,4}} & 0 & Je^{i\varphi_{5,6}} \\ Je^{i\varphi_{6,1}} & Je^{i\varphi_{6,2}} & Je^{i\varphi_{6,3}} & Je^{i\varphi_{6,4}} & Je^{i\varphi_{6,5}} & 0 \end{bmatrix}\begin{bmatrix} a_1 \\ a_2 \\ a_3 \\ a_4 \\ a_5 \\ a_6 \end{bmatrix}=\delta v\begin{bmatrix} a_1 \\ a_2 \\ a_3 \\ a_4 \\ a_5 \\ a_6 \end{bmatrix} \quad (18)$$

By considering the case with $\varphi_{i,j}=-\varphi_{j,i}$, the above matrix can be written as

$$\begin{bmatrix} 0 & Je^{i\varphi_{1,2}} & Je^{i\varphi_{1,3}} & Je^{i\varphi_{1,4}} & Je^{i\varphi_{1,5}} & Je^{i\varphi_{1,6}} \\ Je^{-i\varphi_{1,2}} & 0 & Je^{i\varphi_{2,3}} & Je^{i\varphi_{2,4}} & Je^{i\varphi_{2,5}} & Je^{i\varphi_{2,6}} \\ Je^{-i\varphi_{1,3}} & Je^{-i\varphi_{2,3}} & 0 & Je^{i\varphi_{3,4}} & Je^{i\varphi_{3,5}} & Je^{i\varphi_{3,6}} \\ Je^{-i\varphi_{1,4}} & Je^{-i\varphi_{2,4}} & Je^{-i\varphi_{3,4}} & 0 & Je^{i\varphi_{4,5}} & Je^{i\varphi_{4,6}} \\ Je^{-i\varphi_{1,5}} & Je^{-i\varphi_{2,5}} & Je^{-i\varphi_{3,5}} & Je^{-i\varphi_{4,5}} & 0 & Je^{i\varphi_{5,6}} \\ Je^{-i\varphi_{1,6}} & Je^{-i\varphi_{2,6}} & Je^{-i\varphi_{3,6}} & Je^{-i\varphi_{4,6}} & Je^{-i\varphi_{5,6}} & 0 \end{bmatrix} \begin{bmatrix} a_1 \\ a_2 \\ a_3 \\ a_4 \\ a_5 \\ a_6 \end{bmatrix} = \delta v \begin{bmatrix} a_1 \\ a_2 \\ a_3 \\ a_4 \\ a_5 \\ a_6 \end{bmatrix}, \quad (19)$$

which is the effective Hamiltonian of six coupled site rings. Therefore, based on the scenario described in Eq. S19, we know that the S-matrix can be tuned by manipulating the separation distance between the site rings and the link rings, thereby controlling the coupling strength J. A smaller separation distance results in a stronger coupling strength J, while a larger separation distance leads to a weaker coupling strength J. Additionally, we can tune the coupling phase $\varphi_{i,j}$ between different site rings by manipulating the propagation phase Φ_i of the light wave within the link rings. We can design spatial positions of six site rings so that the phase difference between each adjacent coupling ring is a constant of $\Phi_i = \pi/6, i = 1,2,3,4,5,6$. Therefore, the Hamiltonian equation becomes

$$\begin{bmatrix} 0 & Je^{i\pi/3} & Je^{i\pi/6} & J & Je^{-i\pi/6} & Je^{-i\pi/3} \\ Je^{-i\pi/3} & 0 & Je^{i\pi/3} & Je^{i\pi/6} & J & Je^{-i\pi/6} \\ Je^{-i\pi/6} & Je^{-i\pi/3} & 0 & Je^{i\pi/3} & Je^{i\pi/6} & J \\ J & Je^{-i\pi/6} & Je^{-i\pi/3} & 0 & Je^{i\pi/3} & Je^{i\pi/6} \\ Je^{i\pi/6} & J & Je^{-i\pi/6} & Je^{-i\pi/3} & 0 & Je^{i\pi/3} \\ Je^{i\pi/3} & Je^{i\pi/6} & J & Je^{-i\pi/6} & Je^{-i\pi/3} & 0 \end{bmatrix} \begin{bmatrix} a_1 \\ a_2 \\ a_3 \\ a_4 \\ a_5 \\ a_6 \end{bmatrix} = \delta v \begin{bmatrix} a_1 \\ a_2 \\ a_3 \\ a_4 \\ a_5 \\ a_6 \end{bmatrix} \quad (20)$$

Based on the relationship of $\delta v = \omega - \omega_0$, Eq. S20 can be expressed as

$$\begin{bmatrix} \omega_0 & Je^{i\pi/3} & Je^{i\pi/6} & J & Je^{-i\pi/6} & Je^{-i\pi/3} \\ Je^{-i\pi/3} & \omega_0 & Je^{i\pi/3} & Je^{i\pi/6} & J & Je^{-i\pi/6} \\ Je^{-i\pi/6} & Je^{-i\pi/3} & \omega_0 & Je^{i\pi/3} & Je^{i\pi/6} & J \\ J & Je^{-i\pi/6} & Je^{-i\pi/3} & \omega_0 & Je^{i\pi/3} & Je^{i\pi/6} \\ Je^{i\pi/6} & J & Je^{-i\pi/6} & Je^{-i\pi/3} & \omega_0 & Je^{i\pi/3} \\ Je^{i\pi/3} & Je^{i\pi/6} & J & Je^{-i\pi/6} & Je^{-i\pi/3} & \omega_0 \end{bmatrix} \begin{bmatrix} a_1 \\ a_2 \\ a_3 \\ a_4 \\ a_5 \\ a_6 \end{bmatrix} = \omega \begin{bmatrix} a_1 \\ a_2 \\ a_3 \\ a_4 \\ a_5 \\ a_6 \end{bmatrix} \quad (21)$$

It is clearly shown that the coupling term can be divided into three cases. These three cases correspond to nearest-neighbor (NN) hopping of $Je^{\pm i\pi/3}$, next-nearest-neighbor (NNN) hopping of $Je^{\pm i\pi/3}$, and nearest-nearest-nearest-neighbor (NNNN) hopping of J, respectively. Therefore, the Hamiltonian in the tight-binding model can be expressed as

$$\hat{H} = \sum_{i=1}^{6} \omega_0 a_i^\dagger a_i + J\,exp(i\tfrac{\pi}{3}) a_i^\dagger a_{i+1} + J\,exp(i\tfrac{\pi}{6}) a_i^\dagger a_{i+2} + J a_i^\dagger a_{i+3} + h.c. \quad (22)$$

In the above derivation, we only consider the system in the counterclockwise-spin subspace. Similarly, the effective Hamiltonian in the clockwise-spin subspace is described by

$$\hat{H} = \sum_{i=1}^{6} \omega_0 a_i^\dagger a_i + J\,exp(-i\frac{\pi}{3}) a_i^\dagger a_{i+1} + J\,exp(-i\frac{\pi}{6}) a_i^\dagger a_{i+2} + J a_i^\dagger a_{i+3} + h.c. \quad (23)$$

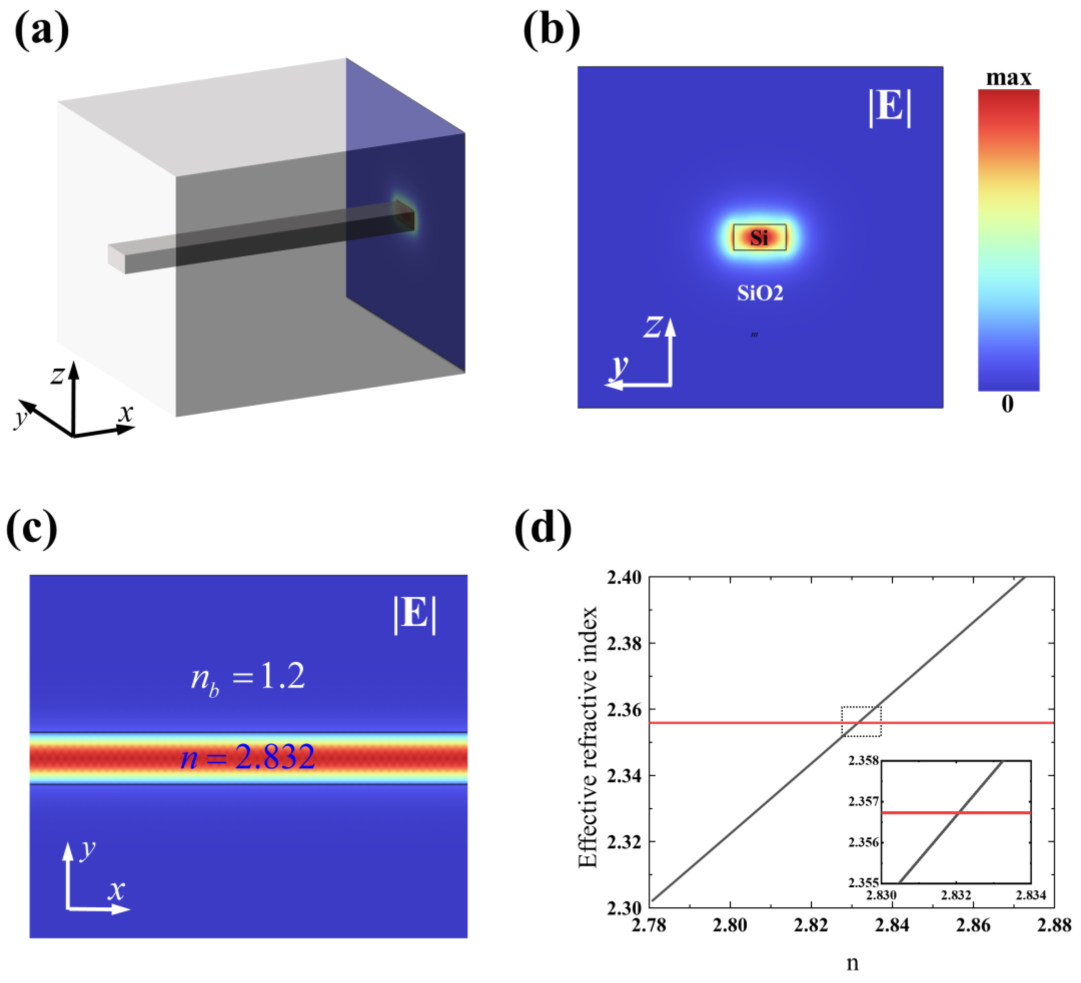

Fig. S6. The Correspondence between effective refractive index of 2D and 3D structures. (a) 3D model of Si waveguide. The waveguide in the middle is made of Si material, surrounded by SiO_2 material. (b) 3D model of Si waveguide is a boundary mode. (c) Propagation mode of 2D model. (d) Correspondence between effective refractive indices of 2D model and 3D mode.

Supplementary Note 3. Numerical results on the performance of optical resonators in three dimensions. Due to the limitation of computing resources, it is impracticable to simulate the entire hyperbolic lattice structure using a full three-dimensional model. Therefore, we consider using an effective two-dimensional model for simulations. Under the approximation of effective medium theory[3,4], the effective permittivity of silicon corresponding to the Si layer is taken by the mode analysis in the finite element method. We consider the structure shown in Fig. S6(a), with an Si

waveguide in the center surrounded by SiO_2 material. Light propagates along the waveguide in the positive x-direction. The refractive indices of Si and SiO_2 are 3.48 and 1.44, respectively. The calculated propagation mode is shown in Fig. S6(b), with an effective refractive index of 2.3567. To enable the effectiveness of the 2D waveguide mode, we consider a 2D waveguide with the refractive index being marked by n, and the environmental refractive index being $n_b = 1.2$, as shown in Fig. S6(c). By adjusting the value of n, the effective refractive index of the 2D waveguide can be obtained, as shown in Fig. S6(d). It can be seen that when n=2.832, the effective refractive index of the 2D mode is consistent with that of the 3D model.

Supplementary Note 4. Numerical results on spatial profiles and robust one-way propagations of topological edge states in vortex-centered hyperbolic lattice model. In this part, we present numerical results on eigenvalues and robust edge propagations in the vortex-centered hyperbolic lattice model. By diagonalizing the Hamiltonian, we can obtain the eigenvalues and eigenstates. Figs. S7(a) and 7(b) present the eigenspectrum and real-space Chern numbers of three-layer structures with φ=π. It is shown that there are lots of hyperbolic edge states in the full eigenspectrum. We find that the nontrivial platforms of real-space Chern numbers appear around eigenenergies of ε=0, showing the existence of hyperbolic topological edge states. While, due the finite size effect, the absolute value of calculated real-space Chern number is smaller than 1. The associated spatial distributions of eigenstates are plotted in Fig. S7(c). It is shown that the probability amplitudes are concentrated in the bulk region for two trivial bulk states with n=1 and n=44. And, two topological edge states with n=45 and n=57 exhibit the significant edge localizations.

Then, we solving coupled model equations Eq. S1. In addition, the input wave packet at the lattice site (m=12) is given by $\psi_{in}(t) = exp(-(t-t_0)^2/100)\sin(\varepsilon_c t)$ with $\varepsilon_c = 0$ and $t_0 = 15$. Fig. S8(a) shows the variation of the wave function when the hyperbolic lattice model without defects. The abscissa axis represents the label of lattice sites, and the ordinate axis represents the time. Fig. S8(b) shows the spatial distribution with t=30, 45 and 50. It can be seen that the input wave propagates unidirectionally along the boundary, manifesting the topological property of hyperbolic edge states.

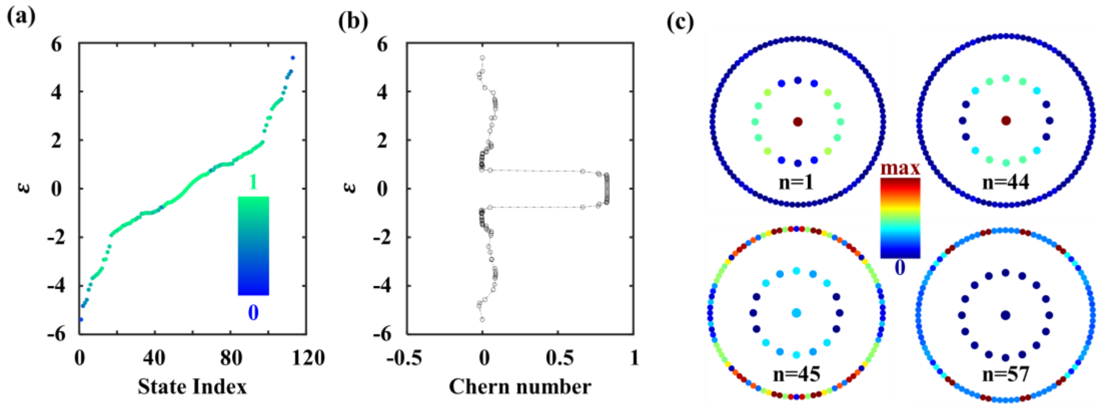

Fig. S7. (a) and (b) The eigenspectra and real-space Chern numbers of the three-layer face-centered hyperbolic lattice model with φ equaling to π. (c) Spatial distributions of hyperbolic eigenstates with n=1, 44, 45, 57. Top and bottom images correspond to bulk and edge states, respectively.

Next, we add a defect at site (m=52) by decoupling it from the rest of the lattice sites. By solving Eq. S1, we obtained the numerical results of wave propagation with the existence of a defect, as shown in Figs. S8(c) and 8(d). It can be observed that light circumvented the defect and continued to propagate forward, proving that our designed structure possesses topologically protected edge states.

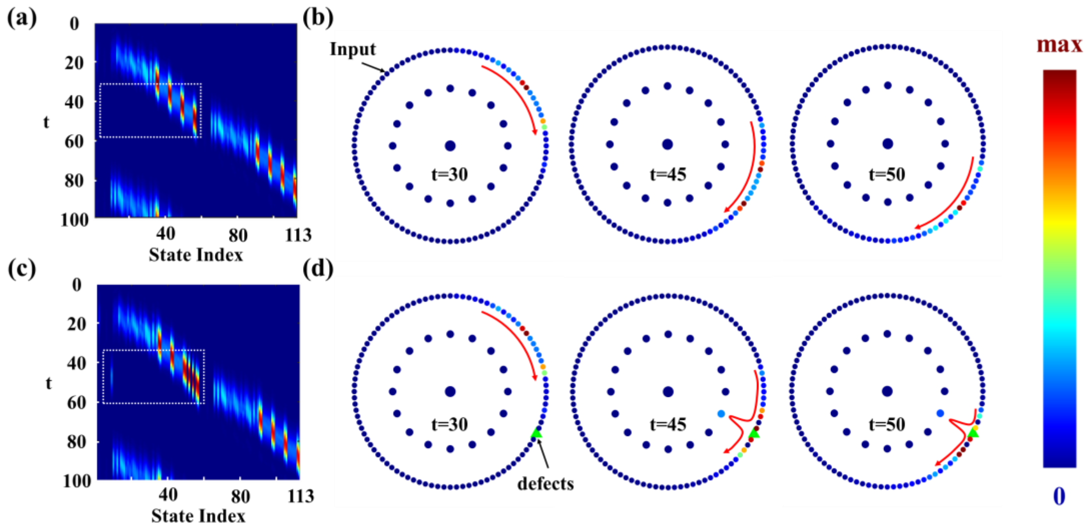

Fig. S8. (a) and (c). The variation of the wave function in the time domain with and without defect hyperbolic lattice model. (b) and (d). Spatial distributions at different times with and without defect hyperbolic lattice models.

Supplementary Note 5. Numerical results of transmission spectra for the hyperbolic photonic topological insulators. Using the finite element method, we calculate the transmission spectra of face-centered and vertex-centered topological photonic topological insulators (shown in Figs. S9(a) and 9(b)). It is shown that the input light can propagate along the CPS and APS channels, respectively. The light propagating along CPS will meet the defect, while the light propagating along APS cannot meet the defect. Figs. S9(c) and 9(d) show the calculated transmission spectra of clockwise and counterclockwise ports for the face-centered structure under excitations of CPS and APS, respectively. Figs. S9(e) and 9(f) show the calculated transmission spectra of clockwise and counterclockwise ports for the vertex-centered structure under excitations of CPS and APS, respectively. The shaded area corresponds to the region of topological edge modes. By comparing these two figures, we can see that the transmission spectra are almost identical, indicating that the structure can support topologically protected edge modes.

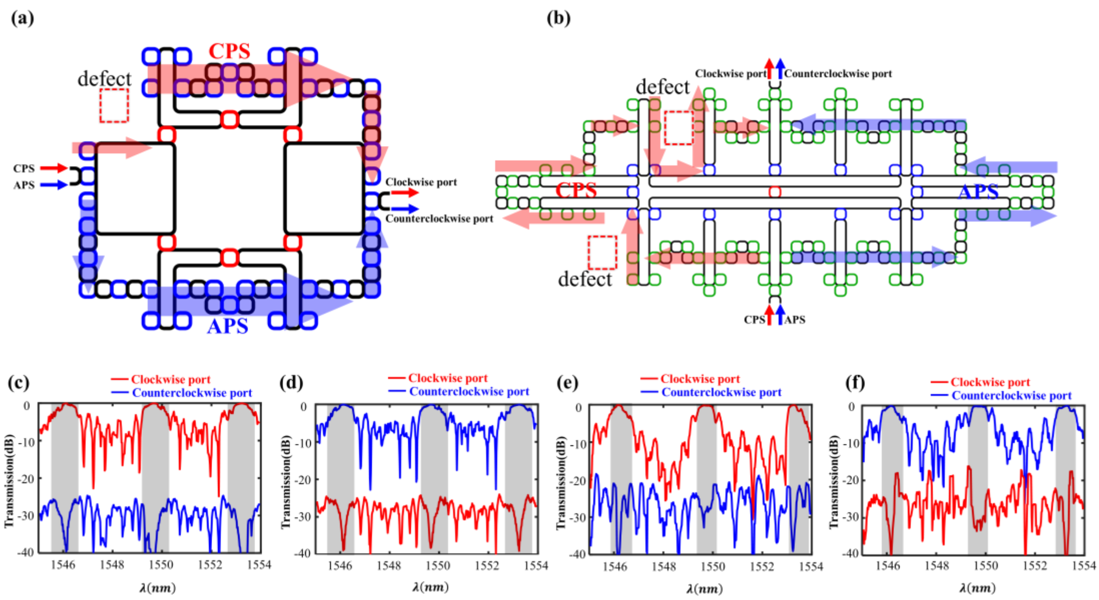

Fig. S9. The transmission spectra of the face-centered hyperbolic topological structure. (a) The schematic diagram of the face-centered hyperbolic topological photon structure with defects. (b) The schematic diagram of the vertex-centered hyperbolic topological photon structure with defects. (c) and (d). Simulated transmission spectra of clockwise and counterclockwise ports for the face-centered structure by exciting CPS and APS. (e) and (f). Simulated transmission spectra of clockwise and counterclockwise ports for the vertex-centered structure by exciting CPS and APS.

Supplementary References

1. Hafezi, M., Demler, E. A., Lukin, M. D. & Taylor, J. M. Robust optical delay lines with topological protection. *Nat. Phys.* **7**, 907–912 (2011).
2. Leykam, D., Mittal, S., Hafezi, M. & Chong, Y. D. Reconfigurable topological phases in next-nearest-neighbor coupled resonator lattices. *Phys. Rev. Lett.* **121**, 023901 (2018).
3. Hammer, M. & Ivanova, O. V. Effective index approximations of photonic crystal slabs: a 2-to-1-D assessment. *Optical and quantum electronics* **41**, 267-283 (2009).
4. Qiu, M. Effective index method for heterostructure-slab-waveguide-based two-dimensional photonic crystals. *Appl Phys Lett.* **81**, 1163–1165(2002).
